# Diabetes and calcific aortic valve disease: controversy of clinical outcomes in diabetes after aortic valve replacement

**DOI:** 10.3389/fendo.2025.1577762

**Published:** 2025-07-30

**Authors:** Feng Liu, Haipeng Cai

**Affiliations:** Department of Cardiology, Taizhou Central Hospital (Taizhou University Hospital), Taizhou, China

**Keywords:** calcific aortic valve disease, diabetes, transcatheter aortic valve replacement, surgical aortic valve replacement, deterioration of bioprosthetic aortic valve

## Abstract

Calcific aortic valve disease (CAVD) is a progressive disease, of which the 2-year mortality is >50% for symptomatic aortic valve stenosis unless transcatheter aortic valve replacement (TAVR) or surgical aortic valve replacement (SAVR) is performed promptly. The prevalence of diabetes among CAVD has increased rapidly in recent years. The combination of diabetes with its cardio-renal and metabolic comorbidities, such as hypertension, hyperlipidemia, chronic kidney disease, and ageing, accelerated the progression of CAVD and increased the subsequent needs for aortic valve replacement. Clinical data regarding the impact of diabetes on outcomes of patients undergoing TAVR or SAVR have exhibited inconsistent results. Compared with non-diabetes, the short-term mortality after TAVR was not significant in diabetes, while the mid-term mortality differed from different cohorts. Although there were worse mid-term and long-term mortalities after SAVR in diabetes, the short-term mortality in diabetes was disputable. As for complications, there were common worse manifestations with coronary heart disease, acute kidney injury, heart failure, and systemic inflammatory response syndrome in diabetes undergoing TAVR or SAVR. Moreover, diabetes was one of the risk factors for deterioration of bioprosthetic aortic valves, leading to increased long-term mortality. Based on the efficacy for CAVD and atherosclerotic cardiovascular disease, glucose-lowering medications might have potential to inhibit deterioration of bioprosthetic aortic valves independent of glucose control.

## 1 Introduction

Calcific aortic valve disease (CAVD) is a progressive disease that has been occurring with rapidly increasing morbidity because of the ageing of the population (1). With the progression of aortic valve stenosis (AVS), the extent of cardiac damage gradually increased from left ventricle to right ventricle (2). Although mortality did not increase when AVS was asymptomatic, the 2-year mortality was more than 50% for patients with symptomatic stenosis (3, 4). Emerging evidence has indicated that CAVD was an active and regulable pathological process in which the risk factors, such as diabetes, hypertension, and hyperlipidemia, were similar to those of other cardiovascular diseases (5, 6). However, lipid-lowering therapy with atorvastatin, simvastatin and ezetimibe, or rosuvastatin did not prevent the progression of CAVD (7–9). There are currently no effective pharmacotherapies to retard or reverse the progression of CAVD. Transcatheter aortic valve replacement (TAVR) and surgical aortic valve replacement (SAVR) are the only effective treatments for end-stage CAVD.

**Figure 1:**
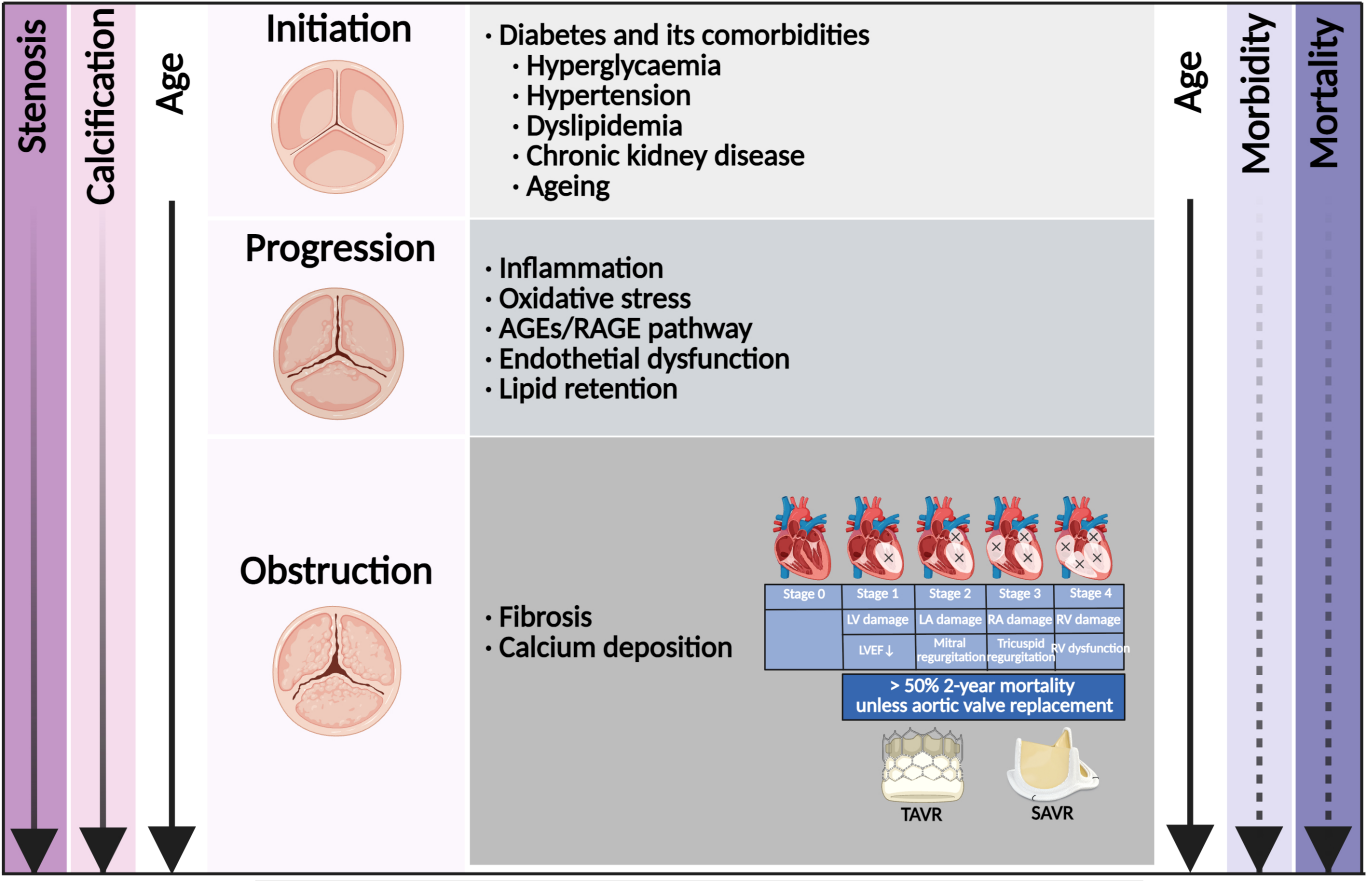
Risk factor and time course of diabetes concomitant to calcific aortic valve disease. Shown is the relationship among disease stage, risk factor, molecular link, valve anatomy, stage of cardiac damage, and the age of the patient. The morbidity of aortic valve stenosis (dashed line) increased rapidly with age. Once in symptomatic stenosis, the mortality of aortic valve stenosis (solid line) increased rapidly. Once with cardiac damage, aortic valve replacement was the only effective treatment. AGEs, advanced glycation end products; RAGE, receptor for AGEs; LV, left ventricle; LA, left atrium; RV, right ventricle; RA, right atrium; LVEF, left ventricular ejection fraction; TAVR, transcatheter aortic valve replacement; SAVR, surgical aortic valve replacement.

Patients with diabetes were at increased risk of developing various cardiovascular diseases, including coronary artery disease (CAD), stroke, peripheral artery diseases, and CAVD (10). According to large-scale retrospective observations worldwide, the prevalence of diabetes in CAVD ranged from 11.4% to 31.6%, and increased by almost 50% in the recent decade (11–14). It was worth noting that the prevalence of diabetes in CAVD undergoing TAVR or SAVR also increased rapidly in recent years (13–16). Diabetes stood as a major risk factor for developing hypertension, hyperlipidemia, chronic kidney disease (CKD), and ageing (17), which were all associated with the initiation of CAVD (**Figure 1**) (18). The combination of diabetes with these cardio-renal and metabolic comorbidities accelerated the progression of CAVD and increased the subsequent needs for aortic valve replacement (AVR). The underlying mechanism of diabetes and its comorbidities involved endothelial dysfunction, immune cell infiltration, oxidative stress, lipid retention, as well as subsequent osteogenic and myofibroblastic differentiation of valvular interstitial cells (VICs) and eventual calcification (18, 19). Once fibrosis and calcification of the aortic valve initiated, the majority of patients developed AVS progressively (20).

Diabetes was generally considered as an adverse factor in patients with cardiovascular diseases needing surgical or invasive interventions. The impact of diabetes on AVR manifested with various complications, such as CAD, acute kidney injury (AKI), heart failure, and systemic inflammatory response syndrome (SIRS). However, clinical data regarding the impact of diabetes on outcomes of patients undergoing AVR have exhibited inconsistent results. The short-term mortality after SAVR was significantly lower in diabetes according to the Spanish National Hospital Discharge Database, while another Spanish study found no difference in short-term mortality between diabetes and non-diabetes patients (14, 21). Diabetes was found as a risk factor for mid-term mortality after TAVR in a meta-analysis including 64 studies (22). However, according to the VARC-2 criteria, diabetic patients did not have increased mid-term mortality after TAVR compared with non-diabetic patients (23). There is currently no article that has summarized the influence of diabetes on clinical outcomes after AVR.

In this review, we present the prevalence of diabetes in CAVD. Then, we discuss the underlying mechanisms of diabetes and its comorbidities in CAVD. Most importantly, we discuss the controversies of clinical outcomes in diabetic patients undergoing TAVR or SAVR. Finally, we summarize updated knowledge about the influence of diabetes on the deterioration of bioprosthetic aortic valve (BAV).

## 2 The prevalence of diabetes in CAVD

With the ageing of the population, the incidence rate of diabetes concomitant to AVS has increased rapidly by years (**Table 1**). The CURRENT AS registry, enrolling 3,815 consecutive patients with CAVD in Japan between 2003 and 2011, showed that 11.4% of patients had concomitant diabetes (11). The multi-central, prospective, observational PRIMID AS study, conducted in 10 hospitals in the United Kingdom between 2012 and 2014, showed that 14.4% of patients with moderate to severe AVS had concomitant diabetes (12). Another study, performed in the Swedish population-based cohort study, showed that the prevalence of diabetes was 15.8% in severe AVS (24). In recent decades, the incidence of AVR increased rapidly in CAVD with diabetes. A retrospective analysis of patients with SAVR between 1987 and 2016 in Netherlands revealed that the prevalence of diabetes has increased from 7.8% to 17.9% over three decades (15). Likewise, the retrospective data from Spanish cohorts (2001–2015) showed that the prevalence of diabetes increased significantly from 16.7% to 23.5% in patients undergoing SAVR (14). The MedPAR file, reporting trends in demographic characteristics associated with isolated AVR in the United States, showed that the prevalence of diabetes increased from 19.7% to 31.6% between 2009 and 2015 in the SAVR cohort and increased from 34.2% to 36.8% between 2012 and 2015 in the TAVR cohort (13). According to the data from the National Inpatient Sample between 2012 and 2017, hospitalizations of diabetes undergoing TAVR increased from 0.97 to 7.68/100,000 adults (16). In the Danish nationwide registers, the prevalence of diabetes undergoing TAVR significantly increased from 14.2% in 2008–2010 to 19.4% in 2017–2018 (25). Overall, with the increased prevalence of diabetes undergoing AVR in recent years, the influences of diabetes on clinical outcomes after TAVR or SAVR have been getting more and more attention.

**Table 1:** The Prevalence of Diabetes in CAVD.

Study type	Characteristic	Population	Conclusion	Ref.
Retrospective	CURRENT AS	3,815	11.4% of CAVD had concomitant diabetes.	(11)
Prospective	PRIMID AS	174	14.4% of patients with moderate to severe AVS had concomitant diabetes.	(12)
Retrospective		777	The prevalence of diabetes was 15.8% in severe AVS.	(24)
Retrospective		4,404	The prevalence of diabetes has increased from 7.8% to 17.9% over three decades, in Netherlandish patients undergoing SAVR.	(14)
Retrospective	SNHDD	78,223	The prevalence of diabetes increased significantly from 16.7% to 23.5% in Spanish patients undergoing SAVR.	(15)
Retrospective	MedPAR	233,660	The prevalence of diabetes increased from 34.2% to 36.8% between 2012 and 2015 in TAVR cohort.	(13)
Retrospective	NIS	428,427	The hospitalizations of diabetes undergoing TAVR increased from 0.97 to 7.68/100,000 patients.	(16)
Retrospective		6,097	The prevalence of diabetes undergoing TAVR increased from 14.2% in 2008-2010 to 19.4% in 2017-2018.	(25)

CAVD, calcific aortic valve disease; AVS, aortic valve stenosis; SAVR, surgical aortic valve replacement; TAVR, transcatheter aortic valve replacement; Ref., reference; CURRENT AS, Contemporary outcomes after sURgery and medical tREatmeNT in patients with severe Aortic Stenosis; PRIMID AS, PRognostic Importance of MIcrovascular Dysfunction in Aortic Stenosis; SNHDD, Spanish National Hospital Discharge Database; MedPAR, Medicare Provider Analysis and Review; NIS, National Inpatient Sample.

## 3 Risk factor and molecular link for CAVD in diabetes

### 3.1 Hyperglycemia

**Figure 2:**
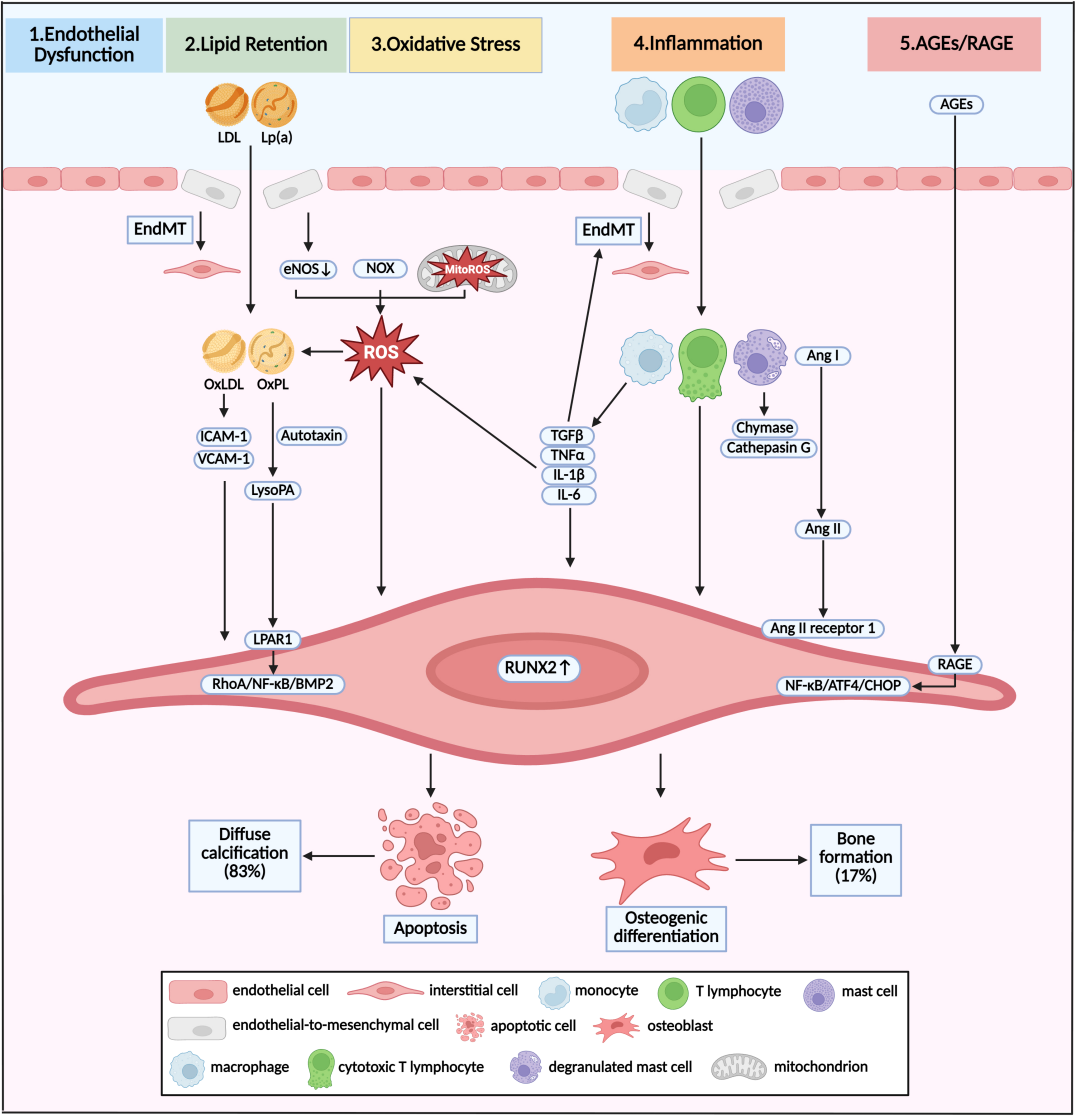
Pathway and molecular link between diabetes and calcific aortic valve disease. Pathological mechanism of initiation and progression of CAVD in diabetes was shown as crosstalk of various pathways. Different stimuli induced EndMT and broke the endothelial barrier, resulting in infiltration of lipoproteins and immune cells. This infiltration was accompanied by the overproduction of ROS via dysregulation of eNOS, and accumulation of NOX and MitoROS. Oxidative stress could promote the formation of OxLDL and OxPL, which induced osteogenic differentiation of VICs, and eventual bone formation. Apoptosis of VICs and subsequent diffuse calcification were induced by infiltrated macrophages, T lymphocytes and mast cells via direct interaction, activation of Ang II, and secretion TGFβ, TNFα, IL-1β, and IL-6. Increased circulating AGEs induced the pro-osteogenic reprogramming via RAGE/NF-κB/ATF4/CHOP pathway. Diffuse calcification accounted for approximately 83% of all calcification deposits, while bone formation accounted for the other 17%. LDL, low-density lipoprotein; Lp(a), lipoprotein (a); EndMT, endothelial-to-mesenchymal transition; ROS, reactive oxygen species; eNOS, endothelial nitric oxide synthase; NOX, nicotinamide adenine dinucleotide phosphate oxidase, MitoROS, mitochondria-generated ROS; OxLDL, oxidized LDL; OxPL, oxidized phospholipids; Ang, angiotensin.

Diabetes is a chronic disease characterized by hyperglycemia and manifested by various cardiovascular diseases. Recent research has found that a hyperglycemia-simulating environment attenuated experimentally induced osteogenic differentiation of cultured human VICs (26). To further mimic the events of aortic valve tissue in diabetic conditions, chronic hyperglycemia was assessed in valvular endothelial cells (VECs) and VICs via a gelatin methacrylate 3-dimension model (27). The gene expressions of MCP-1 and IL-1β were increased both in VECs and VICs after high-glucose treatment for 14 days, exhibiting changes of extracellular matrix (ECM) remodeling and inflammation (27). However, in another dynamic three-dimension aortic valve leaflet model using a software-governed bioreactor system with controlled pulsatile flow, hyperglycemia did not exhibit any impact on fibrosis or calcification on the aortic valve (28). *In vivo*, LDLR^-/-^ApoB^100/100^ mice fed with diabetogenic diet had higher incidence of aortic valve incrassation and stenosis in comparison with normal chow (29). This controversial relationship between hyperglycemia and VIC osteogenesis indicated that there were other complicated mechanisms of diabetes on developing CAVD beyond sole hyperglycemia. Exposure to hyperglycemia in diabetes rapidly accelerated circulating advanced glycation end product (AGE) formation (30). Extracellular AGEs modified global tissue structure and function by binding to the receptor for AGEs (RAGE) (31). In a study on 76 patients undergoing AVR, both the plasma and valvular levels of AGEs were increased in patients with diabetes (32). Overexpression of valvular AGEs was associated with increased mean transvalvular pressure gradient, and overexpression of plasma AGEs was associated with aortic valve area and max transvalvular pressure gradient (32). In an animal model of CAVD, RAGE deficiency attenuated morphometric infiltration, calcification, and AGE accumulation in the aortic valve (33). *In vivo*, the knockdown of RAGE in high-cholesterol diet-fed ApoE^-/-^ mice attenuated the expression of RUNX2 mRNA via NF-κB/ATF4/CHOP pathway (**Figure 2** Panel 5) (34).

### 3.2 Diabetic complications

#### 3.2.1 Hypertension

Hypertension was the most common complication of diabetes, and 40–60% patients with diabetes would develop abnormal blood pressure or hypertension sooner or later (35). Patients with diabetes developed increased arterial resistance caused by vascular remodeling and increased circulating volume caused by hyperglycemia, both of which elevated blood pressure (36). Approximately 70% of the patients with AVS had concomitant hypertension (37). In a large-scale clinical observation involving a 5.4-million population without known valvular heart disease, long-term exposure to elevated blood pressure (median follow-up of 9.2 years) was associated with an increased risk of AVS (38). Specifically, each 20-mmHg increase in systolic blood pressure was associated with a 41% higher risk of AVS (38). VECs were the first cells to be affected by hemodynamic changes. There were pieces of evidence finding that hypertension could accelerate the progression of CAVD by hemodynamic flow disturbance, which could cause mechanical damage to the VECs, especially on the aortic side (39, 40). Under exposure to circulating stimulants, VECs could differentiate into mesenchymal valve progenitor cells, a precursor of VICs, in a process called endothelial-to-mesenchymal transition (EndMT) (19). A relatively high rate of EndMT led to a destruction of the endothelial barrier due to the loss of adherent junction (**Figure 2** Panel 1) (41). Lymphocyte and macrophage infiltrated into the aortic valve through a destroyed endothelial layer and secreted various procalcific and proinflammatory cytokines (42). TGFβ and IL-1β would in turn stimulate the EndMT of VECs (43, 44). When those pathological factors constantly existed in the aortic valve, endothelial-derived VICs could differentiate into the osteoblastic phenotype (19). Once the osteogenesis has been launched, RUNX2 served as the marker of calcification. The current hypotheses suggested that the activation of RUNX2 involved activated STAT3 or STAT5, which, due to activating inflammatory signaling, translocated into the nucleus and bound onto STAT binding sites in the promoter region of RUNX2, leading to the recruitment of additional transcription factors, co-transcription factors, and chromatin remodelers (45, 46).

#### 3.2.2 Hyperlipidemia

Abnormal lipids metabolism was one of the major comorbidities in diabetes, which has been recognized as a hallmark in the early stage of CAVD and could be detected long before calcium deposits by PET-CT (47). A genome-wide meta-analysis of 11.6 million variants in 10 cohorts, involving 653,867 European ancestry participants, supported a causal contribution of lipoprotein (a) (Lp(a)), apolipoprotein B, and low-density lipoprotein (LDL) to AVS (48). In the Global Lipids Genetics Consortium, which included 188,577 participants, the odds ratio for developing AVS per unit increase in lipid parameter was 1.52 for LDL (49), indicating that LDL-lowering medication might be effective in prevention of CAVD. However, three large-scale randomized clinical trials (RCT) failed to illustrate any significant benefit of LDL-lowering medication with statins on the prevention of AVS (7–9), indicating that further studies were needed to seek the association of other lipid indexes and CAVD. Accumulation of reactive oxygen species (ROS) promoted the transformation of LDL to oxidized LDL (OxLDL) and the transformation of Lp(a) to oxidized phospholipids (OxPL), which have been proven to facilitate the osteogenic differentiation of VIC *in vitro* study (**Figure 2** Panel 2) (50, 51). OxLDL increased the expression of cell adhesion molecules, including ICAM-1 and VCAM-1, which consequently promoted RUNX2 expression and calcific remodeling in the aortic valve (52, 53). Liquid chromatography–tandem mass spectrometry demonstrated that lysophosphatidic acid (LysoPA), the decomposition production of OxPL catalyzed by autotaxin, was overexpressed in calcified leaflets in comparison with normal leaflets (54). Further study has shown that OxPL and LysoPA could accelerate the osteogenic differentiation of VICs by binding to LysoPA receptor 1 (LPAR1), levels of which were also increased in calcified leaflets (55). Inhibition of LPAR1 decelerated the progression of AVS and calcium deposits in the aortic valve (56). LPAR1 could instigate a pro-calcific gene program in VICs via RhoA/NF-κB/BMP2 activation (56).

#### 3.2.3 Chronic kidney disease

As one of the major complications of diabetes, approximately 30–40% of patients with diabetes developed diabetic nephropathy eventually (57), which progressively caused CKD and was associated with increased mortality (58). The prevalence of CAVD ranged from 28% to 85% in patients with CKD (59). Even in stage 2 and 3 CKD, >30% of the patients were found to have detectable aortic and/or mitral valve calcification (60). Moreover, functional AVS was found in 9.5% of patients with CKD in comparison with 3.5% of the general population (61). Even when taking account of age, race, sex, diabetes, and hypertension, patients with CKD had a 1.2- to 1.3-fold increased risk of CAVD (61). Multiple mediators in CKD, including hyperphosphatemia, calcium–phosphate product, parathyroid hormone, and systematic inflammation, have been identified as risk factors of calcium deposition in the aortic valve (62). By conducting single-cell RNA sequencing with aortic valve leaflets excised from CAVD patients, VICs (72.64%) accounted for a major proportion among all cell types, followed by monocytic cells (19.52%), lymphocytes (6.23%), VECs (1.28%), and mast cells (0.33%) (63). Immunohistochemistry staining showed chronic immune cells infiltration on calcified valve leaflets, involving CD68+ macrophages, CD3+ T lymphocytes, and mast cells, while only few unactivated immune cells were exhibited in healthy valve leaflets (**Figure 2** Panel 4) (64, 65). Monocytes and macrophages activated the RUNX2 overexpression in VICs through secretion of TGFβ, TNFα, IL-1β, and IL-6 (66). Moreover, the release of extracellular vesicles from macrophages induced diffuse calcification due to the release of apoptotic bodies by VIC apoptosis (67). Likewise, the infiltrated CD8+ cytotoxic lymphocytes in diseased aortic valve induced apoptosis of VICs by direct interaction (68). The bulk of mast cells have been activated to degranulate and release chymase and cathepsin G in the calcified aortic valve, which both could convert angiotensin I to angiotensin II (69). Notably, exposure of VICs to angiotensin II promoted the expression of RUNX2, by binding to the angiotensin II receptor 1 (70). Accordingly, exposure of ApoE^-/-^ mice to high-dose angiotensin II contributed to myofibroblastic differentiation of VICs and eventual aortic valve leaflet thickening (71).

#### 3.2.4 Ageing

Ageing was a powerful independent risk factor for degenerative aortic valve disease. Diabetes was closely associated with ageing and was a major risk factor for ageing-associated cardiovascular diseases (72). Aortic valve calcification used to be viewed as a degenerative process, where calcification was thought to be the consequence of physiological ageing (5). Interestingly, a renewed characterization of the ageing aortic valve has emerged in recent years. Many prior studies that examined “healthy” elderly valves actually involved valve leaflets with calcification (73, 74), meaning that those data could not be used to define the characteristics of a normal, ageing valve. That was because the majority of samples in these references had come from individuals over 60 years old, and approaches and techniques have significantly evolved. Furthermore, while CAVD was considered a disease of old people, recent demographics showed that CAVD could be detected in the twenties, especially in those suffering from bicuspid aortic valve (75). Thus, diabetes-related pathological ageing probably participated more in the onset and development of CAVD compared with physiological ageing. Accelerated oxidative stress was a common factor in ageing and diabetes (76). Three major sources of ROS were uncoupled nitric oxide synthases (NOS), reduced nicotinamide adenine dinucleotide phosphate oxidase (NOX), and mitochondria-generated ROS (MitoROS) (77). Exposure of VECs to exogenous TNFα and H_2_O_2_ promoted endothelial NOS (eNOS) uncoupling, leading to increases in endogenous superoxide and H_2_O_2_ levels, which could promote ECM remodeling and calcium deposition in aortic valve (**Figure 2** Panel 3) (78). Recent evidence has demonstrated that isoform specific NOX-derived ROS might be involved in the development of CAVD. Intense NOX2 accumulation was found in VIC osteogenesis and in calcified regions of aortic valve leaflets (79). In hypercholesteremic mice, the mRNA level of NOX2 was increased in harvested valve leaflets, while no change was observed for NOX4 (80). Mitochondrion was a critical organelle responsible for both ROS production via end product from oxidative phosphorylation and ROS elimination via mitochondrial superoxide dismutase-mediated dismutation of superoxide (81). Loss of mitochondria was found in aortic valve leaflets excised from patients with CAVD and LDLR^-/-^ mice fed with high-cholesterol chow (82). In cultured human VICs, treatment with Lp(a) promoted VIC osteogenic differentiation accompanied by MitoROS production (83). Our recent study revealed that both β-glycerophosphate acid and TGFβ treatment stimulated MitoROS production and RUNX2 expression in VICs, which was accompanied by decreased mitochondrial biogenesis and mitochondrial dysfunction (82).

### 3.3 Multifactorial interactions

Apart from direct effects of diabetes (e.g., hyperglycemia, AGEs/RAGE pathway, oxidative stress, EndMT, and inflammation), there were multifactorial interactions between those diabetic complications (e.g., hypertension, hyperlipidemia, CKD, ageing), which participated jointly in the initiation and progression of CAVD. Hypertension-induced hemodynamic shear stress on the aortic side induced endothelial dysfunction and hampered barrier function, which exacerbated lipid deposition in an aortic valve under a hyperlipidemic condition (84). CKD caused hypertension through an interplay of factors, including water–sodium retention, renin–angiotensin system overactivation, and endothelial dysfunction (85), which were common pathological factors of CAVD. Hyperglycemia represented a key cellular stress in the kidney by altering cellular metabolism in endothelial cells and podocytes (86). Thereafter, increased oxidative stress and activation of inflammatory pathways caused progressive kidney function decline and fibrosis (86). Hyperlipidemia was associated with low-grade systemic inflammation, which might lead to insulin resistance, insulin deficiency, and consequent hyperglycemia (87). As two of the subsets of metabolic syndrome, hyperlipidemia and hyperglycemia jointly promoted CAVD via oxidative stress and chronic inflammation (84).

## 4 Diabetes in transcatheter aortic valve replacement

**Figure 3:**
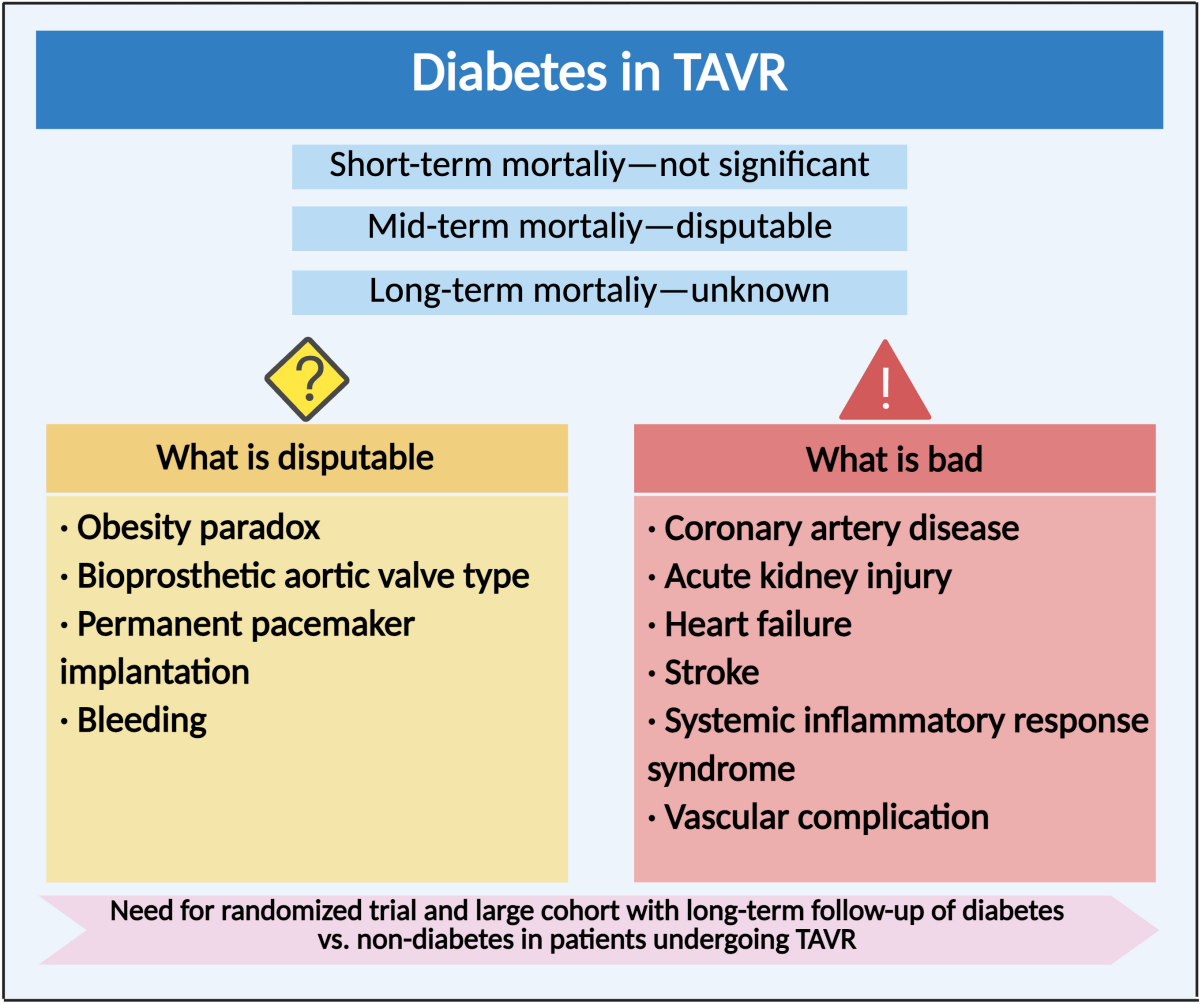
Clinical outcome and complication of diabetes in transcatheter aortic valve replacement. Compared with non-diabetes, the impact of diabetes on clinical outcomes and various complications after TAVR.

TAVR was a percutaneous treatment option for symptomatic severe aortic valve stenosis, especially among patients at high surgical risk (88). The prevalence of diabetes was up to a third of cases in TAVR patients (89–91). However, the association between diabetes and outcomes after TAVR procedure remained controversial (**Table 2**). Some studies described similar rates of complications (90, 92), while others reported higher (89, 91) or even lower 1-year mortality rates (93, 94). This controversy was represented in mortality risk prediction scores, which were based on data from surgical patients. STS-PROM score included diabetes as a risk factor (95). However, logistic EuroSCORE did not include diabetes as a risk factor (96), whereas EuroSCORE II only included insulin-treated diabetes (97). EuroSCORE II was the most accurate risk score with slight underestimation of actual mortality, whereas STS-PROM score and logistic EuroSCORE overestimated observed mortality (98). A meta-analysis including 64 studies with a total of 38,686 patients found that diabetes was associated with increased 1-year mortality after TAVR (22). Moreover, although insulin-treated diabetes was not associated with adverse outcome compared with orally treated diabetes, elevated HbA1c levels might be associated with increased mortality during long-term follow-up (99). Stress hyperglycemia ratio has recently been recognized as an accurate biomarker that represented true hyperglycemia status (100). In a prospective single-center study with a median follow-up of 3.9 years, stress hyperglycemia ratio was linearly associated with all-cause mortality and cardiovascular mortality in patients undergoing TAVR (101). However, according to the VARC-2 criteria, there were no significant differences of 30-day and 2-year mortality in patients undergoing TAVR between diabetes and non-diabetes (23, 99). Moreover, in a propensity matched analysis of multicentral registry including data from >12,000 patients undergoing transfemoral TAVR, diabetes was not associated with worse outcomes within the first year after TAVR, which underscored the safety of TAVR treatment in diabetes patients (98). Similarly, according to the data from the Spanish cohorts, diabetic patients undergoing TAVR did not exhibit increased in-hospital mortality compared with non‐diabetic patients (94). Further study has found some relationship between diabetes and TAVR in a specific cohort. An observational study of all consecutive patients treated with a transfemoral TAVR in a single-center cohort revealed that male patients with diabetes had significantly higher 3-year mortality compared with males without diabetes and there was no difference in 3-year mortality for female patients with and without diabetes, indicating gender-dependent association between diabetes and mortality after TAVR (102). Nevertheless, another observational study demonstrated that no influences of diabetes presence on the risk of 30-day and 1-year mortality after adjustment for age and gender (103). Anyway, in a *post-hoc* analysis of the PARTNER trial, diabetic patients were noted to have decreased 1-year mortality when treated with TAVR compared to SAVR (93). Overall, the short-term mortality after TAVR was not significant between diabetes and non-diabetes, while the mid-term mortality remained controversial. Taken together, these observations might tend to favor a transcatheter approach when either approach would be a reasonable option, particularly in those with diabetes. There is a need for RCT and large cohorts with long-term follow-up of diabetes vs. non-diabetes in patients undergoing TAVR (**Figure 3**).

**Table 2:** Diabetes in TAVR.

Study type	Characteristic	Population	Conclusion	Ref.
Retrospective	SNHDD	2,141	Diabetes undergoing TAVR did not exhibit increased in-hospital mortality compared with non‐diabetes.	(94)
Retrospective		586	Diabetes was not associated with an increased 30-day mortality after TAVR.	(99)
Prospective	VARC-2	443	Diabetes seemed to have similar 30-day and 1-year mortality after TAVR compared with non-diabetes.	(23, 103)
Prospective	OCEAN-TAVI	2,588	Presence of diabetes was significantly associated with higher 2-year mortality after TAVR.	(89)
Retrospective	STS/ACC TVT	47,643	Diabetes was associated with increased 1-year mortality after TAVR.	(91)
Retrospective	TAVIK	2,000	Diabetes was not an independent factor associated with 3-year mortality in TAVR.	(90)
Retrospective	WIN-TAVI	1,012	Insulin-dependent diabetes was not associated with an increased 30-day and 1-year mortality after TAVR.	(92)
Prospective	PARTNER	699	The 1-year mortality after TAVR was lower in diabetes than non-diabetes.	(93)

CAVD, calcific aortic valve disease; VARC-2, Valve Academic Research Consortium 2; OCEAN-TAVI, Optimized transCathEter vAlvular iNtervention-transcatheter aortic valve implantation; STS/ACC TVT, Society of Thoracic Surgeons/American College of Cardiology Transcatheter Valve Therapy; TAVIK, TAVI team Karlsruhe; WIN-TAVI, Women's International Transcatheter Aortic Valve implantation; PARTNER, Placement of Aortic Transcatheter Valves.

### 4.1 TAVR procedure

The risk factors of CAVD, including diabetes, hypertension, and CKD, were more involved in the disease development in patients with tricuspid aortic valves than those with bicuspid aortic valves (104). In asymptomatic moderate-to-severe AVS, patients with tricuspid aortic valves were older and had higher proportion of diabetes, compared with bicuspid aortic valves (104). However, the in-hospital mortality did not differ between transfemoral and transapical access in diabetes undergoing TAVR (105). Transfemoral access was associated with a higher incidence of vascular complications and permanent pacemaker implantation (PPI) implantation than transapical access (105). Especially, transfemoral access for TAVR was associated with higher mortality, acute stroke, AKI, hemodialysis, and percutaneous coronary intervention (PCI) in complicated diabetes with diabetes-related complications than in non-complicated diabetes (105). Although balloon pre-dilatation was not associated with device success or any post-procedural complications in TAVR procedure, there was less diabetes in pre-dilatation group (106). There were overall comparable outcomes between balloon-expandable and self-expanding valve for TAVR (107). In a subgroup analysis of in-hospital mortality, there was no significant difference concerning the type of valve in diabetes (108).

### 4.2 Obesity

The “abnormality” in in-hospital and short-term mortality in diabetes after TAVR might be attributed to diabetes-related obesity. The relationship between body mass index (BMI) and cardiovascular risk prediction was recognized as an “obesity paradox”. On the one hand, obesity has long been established as a risk factor for atherosclerotic cardiovascular disease (ASCVD) (109). On the other hand, increased BMI was found as a protective factor in patients undergoing cardiovascular surgery or intervention (110, 111). Similarly, in comparison with normal-weight patients, patients who were overweight or had obesity had a lower incidence of 30-day mortality, 1-year mortality, and long-term mortality after TAVR (112). Obesity was one of the most common concomitant statuses in patients with diabetes. In patients undergoing TAVR, increased BMI was associated with increased rate of diabetes at baseline (113). However, obesity has been identified as an independent risk factor for vascular complications, PPI, and AKI in patients undergoing TAVR (114, 115). The association between obesity and other post-TAVR complications, including cerebrovascular events, new-onset atrial fibrillation, and myocardial infarction, has received emerging research attention. Therefore, the nuanced influence of obesity on TAVR outcomes necessitated deeper exploration, particularly considering the unique physiological and metabolic profiles inherent to individuals with obesity.

### 4.3 Coronary artery disease

CAD and CAVD were frequently concomitant due to similar risk factors, such as diabetes. About 25% of TAVR recipients have undergone PCI before TAVR in real-world TAVR registries (116). However, coronary artery bypass grafting (CABG) at the time of SAVR has been considered the gold standard in such patients (117). More and more studies pointed to TAVR+PCI as an alternative method (118). Patients with prior CABG had higher rate of diabetes (119), which might be due to higher rate of three-vessel CAD and higher SYNTAX score in diabetes. As for prior PCI, diabetes was associated with increased risk of major adverse cardiovascular events (MACE) (120). However, in stable CAD with diabetes, completeness of PCI either staged or concomitantly with TAVR was similar to undergoing TAVR without PCI concerning the all-cause death and MACE (121). As for acute coronary syndrome after TAVR, the concomitance with diabetes was associated with a higher rate of early mortality among patients undergoing urgent or emergent PCI (122, 123).

### 4.4 Acute kidney injury

Adjusted multivariate Cox regression analyses found that AKI was associated with increased risk of long-term mortality after TAVR (23). A meta-analysis of 64 studies showed that diabetes was associated with increased AKI after TAVR (22). Similarly, based on the data from 410 patients undergoing TAVR, kidney function improvement after TAVR was lower in diabetes than that in non-diabetes (124, 125). Among patients with end-stage renal disease, diabetes was one of the predictors of dialysis and readmission after TAVR (126–128). Diabetes was known to be a cause and prognostic factor for patients on dialysis. The influence of diabetes on kidney function after TAVR was mainly dependent on the baseline glomerular filtration rate and prior blood glucose control. Concomitance with diabetic nephropathy, especially with end-stage renal disease, might be associated with worse outcome after TAVR in diabetic patients.

### 4.5 Heart failure

In TAVR, early findings showed that heart-failure-related death and sudden cardiac death accounted for approximately a third of total deaths and two-thirds of cardiac related deaths (129, 130). Among TAVRs with a newer-generation device including the self-expandable Evolut R/Pro/Pro+ valve and balloon-expandable SAPIEN S3/ULTRA valve, advanced heart-failure-related deaths accounted for 11.6% of total deaths and sudden cardiac death accounted for 7.5% of total deaths (131). Diabetes was independently associated with an increased risk of sudden cardiac death in TAVR (131). The several possible mechanisms were silent myocardial ischemia, QT interval prolongation, diabetic cardiomyopathy, increased arrhythmogenic potential, and hypercoagulability (132). Patients with diabetes were at an increased risk of hospitalization for heart failure at 1-year after TAVR (133, 134). Most of the patients obtained myocardial recovery after TAVR due to decreased cardiac afterload. However, post-TAVR left ventricular ejection fraction recovery was impaired in patients with diabetes (135, 136). Diabetes was associated with elevated left ventricular filling pressure and prior right ventricular dysfunction (137, 138), resulting in more severe heart failure symptoms and more loop diuretic therapy after TAVR (139). These residual myocardial injuries might explain the inferior manifestation of heart failure. The underlying pathophysiologic mechanisms might include changes in vascular homeostasis with diminished nitric oxide and increased ROS levels due to prolonged hyperglycemia, insulin resistance, and hyperinsulinemia, which activated pro-inflammatory pathways that resulted in the progression of atherothrombosis and dysfunction of the myocardium (140). Moreover, diabetes was associated with chronic and new-onset heart failure through neurohormonal dysregulation inducing cardiac fibrosis and decreasing cardiac efficiency (133).

### 4.6 Stroke

Post-procedural stroke was a devastating complication after TAVR and was associated with decreased long-term survival and reduced quality of life (141, 142). The national SWENTRY registry identified diabetes as one of the pre-disposing factors for stroke after TAVR (143). Up to 70% of the stroke was presented with clinically silent stroke or peri-procedural silent brain infarcts, which might be due to subclinical leaflet thrombosis (144). Meta-regression found that diabetes was associated with increased risk of silent brain infarcts (145). In prospective RETORIC trial, diabetes was an independent predictor of event of subclinical leaflet thrombosis (146). This should not seem surprising, as diabetes was independently linked to a higher risk of cerebrovascular disease, thereby reducing the insult threshold required for an ischemic event.

### 4.7 Permanent pacemaker implantation

Intraventricular conduction abnormalities, particularly high-degree atrioventricular block, requiring PPI were one of the major complications after TAVR procedure (147). New-onset left bundle branch block and diabetes independently predicted high-degree atrioventricular block requiring PPI after TAVR and helped to identify patients at risk (148–150). Moreover, post-TAVR PPI was associated with hospitalization of heart failure and myocardial infarction (150). However, there was still a literature finding diabetes as a negative predictor of PPI following TAVR (151). This contradictory result might be affected by confounders not included in the multivariate analysis, such as size of implanted valve and diabetic status.

### 4.8 Systemic inflammatory response syndrome

Previous work has reported that approximately one-third of patients developed an acute inflammatory response within 48 hours after TAVR (152). Therein, severe SIRS developed in approximately 6% of patients undergoing TAVR (153). This SIRS was manifested with significantly elevated levels of inflammatory cytokines (such as IL-6 or IL-8) and C-reactive protein (CRP). The occurrence of SIRS was shown to be a strong predictor of mortality in patients undergoing TAVR (153, 154). Previous study found that the presence of diabetes, increased baseline high sensitivity CRP, and low baseline Th2 cell counts were multivariate predictors of death after TAVR (155). Another study found elevated fasting glucose and CRP level as predictors of increased all-cause mortality after TAVR (156). As mentioned above, diabetes was associated with systematic inflammation in patients with CAVD. Plasma proteomics analysis of the biomarker cohort revealed that IL‐1 receptors, GDF15, and cathepsin D were significantly elevated and that pathways related to inflammatory response were enriched in diabetic CAVD patients (157). Overall, the chronic systemic inflammation status in diabetes would be activated to some extent in TAVR procedure, and the evocative severe SIRS was associated with the mortality.

### 4.9 Bleeding

In order to prevent postoperative thrombosis and delay artificial valve dysfunction, dual antiplatelet therapy was routinely required after TAVR procedure. Long-term bleeding after TAVR was associated with an increased risk of subsequent mortality (158). Previous study found that patients with high bleeding risk after TAVR were more frequently presented with diabetes compared with those with lower bleeding risk (159). Moreover, there were some research studies finding that patients with diabetes had a higher risk of major bleedings compared with those without diabetes after TAVR (91, 160, 161). Nevertheless, there was another research finding no significant differences in major bleeding after TAVR between diabetes and non-diabetes (22). Baseline diabetes was not associated with baseline platelet reactivity levels in TAVR procedure (162). Similarly, there were biases against the role of diabetes on bleeding in other cardiovascular diseases (163). At present, there are no differences in the antiplatelet therapy strategy following TAVR between diabetes and non-diabetes. The absolute benefits of antiplatelet therapy were largely counterbalanced by the bleeding hazard.

## 5 Diabetes in surgical aortic valve replacement

**Figure 4:**
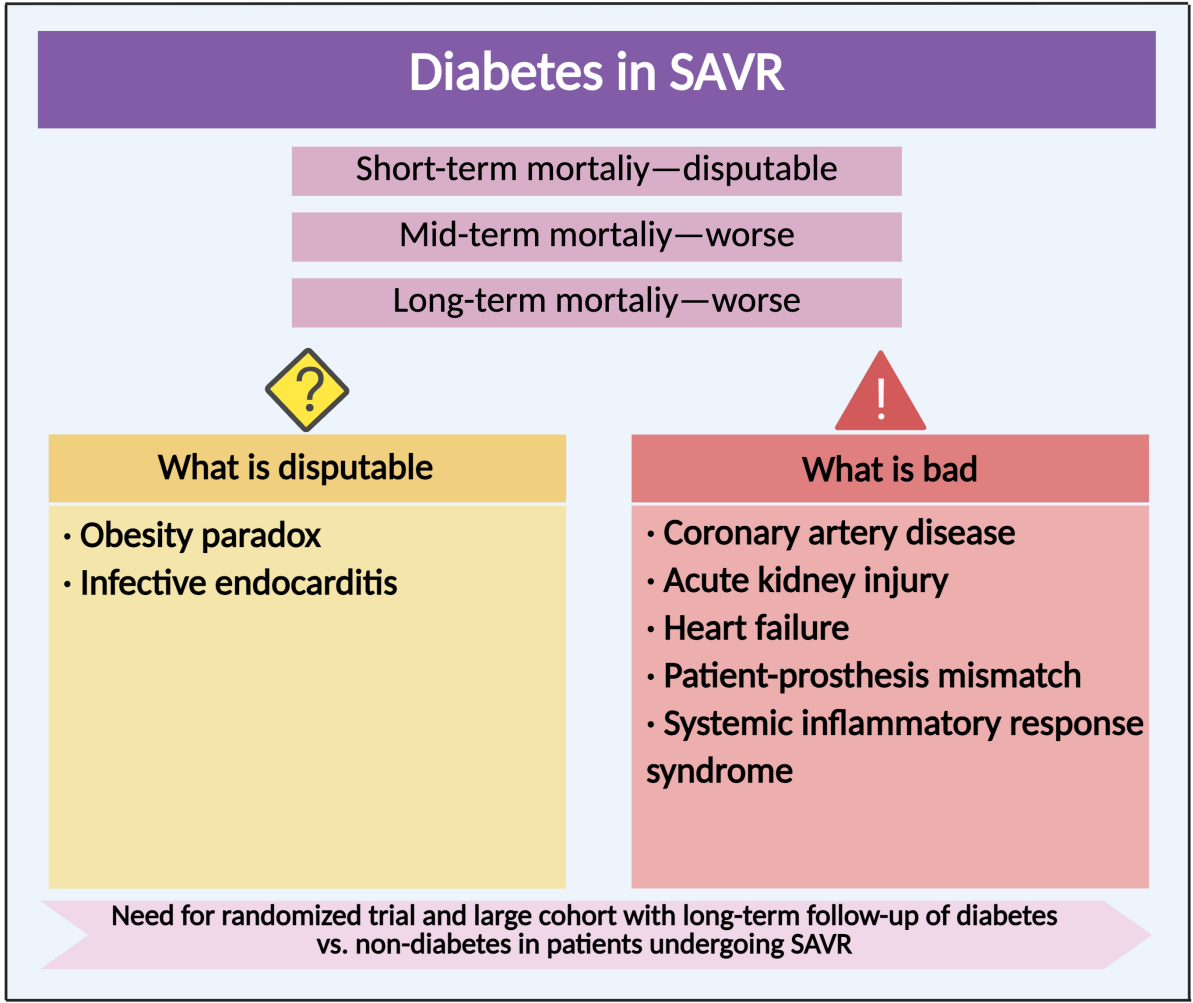
Clinical outcome and complication of diabetes in surgical aortic valve replacement. Compared with non-diabetes, the impact of diabetes on clinical outcomes and various complications after SAVR.

In recent decades, the incidence of bioprosthetic SAVR increased significantly in those with diabetes, which might partly be attributed to an increased prevalence of CAVD and an increased proportion of diseased patients diagnosed as such with ageing of population (14, 164). According to a retrospective study from Spanish cohorts, in‐hospital mortality was significantly lower in diabetes undergoing bioprosthetic SAVR than non-diabetes, which might be multifactorial (**Table 3**) (14). The impact of diabetes on short-term mortality after SAVR still remained controversial; diabetes has been found to be significantly and consistently associated to higher in-hospital mortality in a huge Spanish population after MACE, and there was no difference in 30-day mortality between diabetes and non-diabetes (21). However, the impact of diabetes on mid-term and long-term mortality after SAVR was consistent in several clinical studies. Long-term (5-year and 10-year) mortality was significantly higher in diabetes after SAVR compared with non-diabetes (19.4% vs. 12.9% and 30.3% vs. 23.5%) (165). A *post-hoc* analysis of the PARTNER trial, stratified according to the diabetes status of patients randomly assigned to undergo SAVR, revealed a 1-year mortality rate of 27.4% in diabetes and 23.7% in non-diabetes (93). This association was stronger among insulin-treated diabetes. In-hospital and long-term mortality rates were higher in the subgroup of insulin-treated diabetes compared with the subgroup of non-insulin treated diabetes (165). Insulin-treated diabetes had more comorbidities than non-insulin-treated diabetes and was prone to more revascularization procedures (166). Therefore, the results of mortality after SAVR in diabetes were partially dependent on the baseline diabetic status. Overall, the mid-term and long-term mortality rates after SAVR were higher in patients with diabetes than non-diabetes, while the short-term mortality remained controversial. There is a need for RCT and large cohorts with long-term follow-up of diabetes vs. non-diabetes in patients undergoing SAVR (**Figure 4**).

**Table 3:** Diabetes in SAVR.

Study type	Characteristic	Population	Conclusion	Ref.
Retrospective	SNHDD	78,223	In‐hospital mortality was significantly lower in diabetes undergoing SAVR than non-diabetes in Spain (2001-2015).	(15)
Retrospective	SNHDD	100,854	there was no difference in in-hospital mortality between diabetes and non-diabetes, in patients undergoing SAVR from 2002 to 2014.	(21)
Prospective	PARTNER	699	The 1-year mortality after SAVR was 27.4% in diabetes and 23.7% in non-diabetes.	(93)
Retrospective		1,053	Diabetes was an independent predictor for long-term mortality after SAVR.	(165)

### 5.1 Obesity

Obesity was more prevalent in diabetes undergoing SAVR, which might have contributed to the decrease in in‐hospital mortality (167). The obesity paradox also indeed existed within the realm of SAVR. Post-SAVR complications such as myocardial infarction, stroke, reoperation rates, AKI, new renal failure, requirement of dialysis, and postoperative bleeding were either more frequent in patients with higher BMI or equivalent to their normal BMI counterparts (168, 169). Therefore, the role of obesity in SAVR was disputable, regarding the integrated consideration of mortality and postoperative complications. As obesity was a worldwide problem and surgical techniques were advancing, identification of the underlying causes of the obesity paradox was essential to providing optimal care for patients of all body sizes undergoing SAVR.

### 5.2 Coronary artery disease

Recent retrospective studies revealed that concomitant SAVR and CABG were associated with a significantly higher in-hospital mortality, while there was no additional mid-term or long-term survival risk compared with isolated SAVR (170–172). In patients undergoing concomitant SAVR and CABG, diabetes was associated with 30-day, 180-day, and long-term mortality (170, 173). No RCT focused on the long-term survival of performing a concomitant CABG with SAVR are currently available. Long-term mortality was higher in diabetes vs. non-diabetes, and especially in insulin- vs. non-insulin-treated diabetes regardless of undergoing PCI or CABG (174). Although the long-term mortality was not different in diabetes treated either with PCI or CABG, lower mortality was observed in CABG in the cohort with three-vessel CAD and high SYNTAX score (175). Based on the potential organ-protective and anti-inflammatory effects, a randomized, placebo-controlled clinical trial for efficacy of glucagon-like peptide-1 (GLP-1) receptor agonist exenatide and restrictive versus liberal oxygen supply in patients undergoing CABG with/without SAVR is in progress (176). The researchers intended to determine whether glucose-lowering medication could improve the outcomes in such high-risk patients.

### 5.3 Acute kidney injury

AKI was another serious complication after SAVR and held increased mortality. Diabetes was significantly associated with the development of AKI after SAVR (177, 178). Insulin-dependent diabetes was one of the predictors for post-SAVR renal failure with hemodialysis (179). Kidney recovery after SAVR was more frequent than AKI, which was associated with improved secondary clinical outcomes (180). Diabetes was a negative predictor of kidney recovery after SAVR (125), indicating less reversible kidney injury in diabetes. Compared with patients undergoing SAVR without hemodialysis, patients with chronic renal failure on hemodialysis had diabetes more frequently (181). Moreover, concomitance with diabetes was associated with increased 30-day mortality in chronic dialysis patients after SAVR (182). The conclusions in findings might be related to differences in patients’ controlled hyperglycemia, use of medications, and prior glomerular filtration rate, as diabetes was associated with ischemia and kidney injury.

### 5.4 Heart failure

Although SAVR resulted in significant improvements of pre-existing myocardial impairments, concomitance with diabetes exhibited more residual changes in myocardial structure, contractile function, and blood flow (135, 183). It might be due to more cumulative myocardial injuries in diabetes prior to SAVR and persistent systematic diabetic toxicity after replacement. Therefore, diabetes was one of the independent risk factors of rehospitalization for heart failure after SAVR (184). The initiation and development of myocardial structural/functional abnormalities leading to heart failure in diabetes included multiple mechanisms, which remained incompletely elucidated (185). Nevertheless, they encompassed common systemic factors with CAVD, including hyperglycemia, insulin resistance, excessive production of AGEs, and activation of the renin–angiotensin–aldosterone system (186). Therefore, further studies are needed to explore the potential efficacy of glucose-lowering medications in alleviating both diabetic myocardial and aortic valve injuries.

### 5.5 Patient–prosthesis mismatch

The concept of patient–prosthetic mismatch (PPM) after SAVR referred to the clinical situation in which a normally functioning prosthetic valve did not allow an adequate cardiac output without an excessive gradient across the aortic valve. The prevalence of moderate PPM ranged from 20% to 70% and that of severe PPM ranged from 2% to 20%, respectively (187). Clinical outcomes of patients with mild and moderate PPM were not significantly different to those without PPM, while severe PPM was associated with increased mid-term and long-term mortality after SAVR (188). Diabetes was one of the predictors of the prevalence of PPM in patients undergoing SAVR (189, 190). Diabetes was associated with the occurrence of mild and moderate PPM but did not have a significant effect on the occurrence of severe PPM (191). As PPM was only marginally associated with survival, it was not related to risk of reoperation after SAVR (192).

### 5.6 Systemic inflammatory response syndrome

SIRS developed in 11% of SAVR patients and was associated with a higher mortality after SAVR (153). Although diabetes was not associated with an increased risk of SIRS, severe SIRS had a greater effect on mortality in diabetes (153). Proteomics analysis of plasma from CAVD with diabetes found that IL-1 receptors, GDF15, and cathepsin D as well as the pathways associated with inflammation were significantly elevated (157). This systemic pro-inflammatory response might account for the worse clinical outcomes in diabetes undergoing SAVR.

### 5.7 Infective endocarditis

According to the latest 10-year outcomes of the NOTION trial, the rate of infective endocarditis was similar for both TAVR and SAVR (7.2% vs. 7.4%) (193). Once endocarditis occurred, the all-cause mortality increased rapidly, especially in the SAVR cohort (194, 195). Among patients with endocarditis, the rate of all-cause 1-year or 5-year mortality was higher in the SAVR group than that in the TAVR group (195). In the analysis of pooled statistics of three large RCT, patients with endocarditis after SAVR had diabetes more frequently at baseline than those without endocarditis (195). However, in the PARTNER 1 and PARTNER 2 trials, concomitance with diabetes was not associated with the occurrence of endocarditis after SAVR (194). The baseline clinical characteristics of patient population in different study, including age, previous CABG, and other valvular heart diseases, were disparate. The difference in those factors might account for the differences observed. Further RCT are needed to elucidate whether concomitance with diabetes is associated with the occurrence of endocarditis after SAVR and related mortality.

## 6 Diabetes in bioprosthetic aortic valve deterioration

### 6.1 Bioprosthetic aortic valve deterioration after AVR

Structural valve deterioration (SVD), manifested with leaflet calcification or fibrosis, was one of the pivotal factors limiting the durability of BAV and the prognosis after transcatheter or surgical replacement. Non-structural bioprosthetic valve dysfunction (BVD), defined as any abnormality not intrinsic to the aortic valve, included PPM and paravalvular regurgitation, which occurred at the time of SAVR or TAVR procedure and existed persistently during follow-up, while SVD developed progressively during follow-up. The durability of BAV is becoming a critical problem of TAVR, as this procedure is now considered for younger and lower-risk populations with longer life expectancy (196). In a propensity-matched analysis of intermediate-risk patients (PARTNER 2 and PARTNER 2A), the incidence of SVD at 5 years was 3.9% in TAVR with balloon-expandable SAPIEN 3 vs. 3.5% in SAVR (197). In the PARTNER 3 trial, the incidence of SVD at 5 years was 4.2% in TAVR with SAPIEN 3 vs. 3.8% in SAVR among low-risk patients (196). As for self-expanding CoreValve or Evolut, the CoreValve US High Risk Pivotal and SURTAVI trials found a lower rate of SVD in intermediate- or high-risk patients undergoing self-expanding TAVR vs. SAVR at 5 years (1.82% in TAVR vs. 2.67% in SAVR) (198). The only long-term NOTION trial revealed similar results in comparison between TAVR with CoreValve and SAVR (12.5% in TAVR vs. 13.9% in SAVR) (193). The CHOICE trial compared the first or second generations of SAPIEN with CoreValve in high-risk patients and found superior valve hemodynamic performance for self-expanding valves with lower rate of SVD at 5 years (199). The SMART trial also found that the self-expanding valve was superior to the balloon-expandable valve in the aspect of SVD at 1-year among patients with small aortic annulus (200). The superior performance of the self-expanding valve in SVD might be due to the supra-annular design with better hemodynamic properties. Large RCT with long follow-up are thus needed to compare the durability of different TAVR prostheses, especially among low-risk populations.

### 6.2 Bioprosthetic aortic valve deterioration in diabetes

Although deterioration of BAV has long been considered as a passive degenerative process, emerging studies revealed that active and potentially modifiable mechanisms might also participate in the fibrocalcific process of BAV (201). SVD shared common risk factors and similar pathological process with CAVD. One of the crucial risk factors that have been associated with SVD following TAVR or SAVR was diabetes (197, 198, 201, 202). In the PARTNER 2 trial, diabetes was associated with SVD at 5 years in the SAPIEN 3 TAVR cohort (197). In the prospective study of SVD after SAVR, univariate and multivariate Cox regression analyses found diabetes as one of the risk factors for deterioration of BAV and all-cause mortality (201). In another retrospective study, diabetes was associated with hemodynamic deterioration of BAV, especially at early years (202). Thus, concomitance with diabetes accelerated the deterioration of BAV and restricted the durability of BAV after TAVR or SAVR. The underlying mechanisms of SVD in diabetic conditions might include inflammation, oxidative stress, lipid retention, endothelial dysfunction, and AGEs/RAGE, which were similar to the molecular mechanisms of CAVD concomitant to diabetes as mentioned above. Diabetes was one of the predictors for the prevalence of PPM (189, 190), which in turn caused BVD after AVR. Bioprosthetic valve endocarditis was often associated with morphologic and hemodynamic valve deterioration and might thus lead to SVD (203). One of the prevalent predisposing conditions of prosthetic valve endocarditis was diabetes (204). The retrospective cohort study, conducted in five German cardiac surgery centers, multivariable analyzed 3,143 patients (73.1%) undergoing surgery for native valve endocarditis and 1,157 patients (26.9%) for prosthetic valve endocarditis (205). Patients with prosthetic valve endocarditis presented with higher proportion of diabetes than native valve endocarditis (205). Transcatheter valve-in-valve implantation was an alternative option in inoperable or high-risk patients with severe SVD (206). Age and diabetes were identified as independent predictors of all-cause 30-day mortality in patients with transcatheter valve-in-valve implantation (207). Currently, most of the clinically available BAVs are fabricated from glutaraldehyde-treated heterogeneous aortic valves or bovine/porcine pericardium (208). Previous studies have indicated superior hemodynamic characteristics in bovine pericardial valves compared to porcine valves (209, 210). Moreover, bovine valves were associated with better survival than porcine valves in diabetes (211). However, to date, no vitro or vivo experiments has specifically investigated the influences of diabetes on different bioprosthetic pericardial valves. Diabetes and its complications jointly participated in the deterioration of BAV, leading to increased long-term mortality and various complications after AVR.

### 6.3 Glucose-lowering medications in bioprosthetic aortic valve deterioration

**Figure 5:**
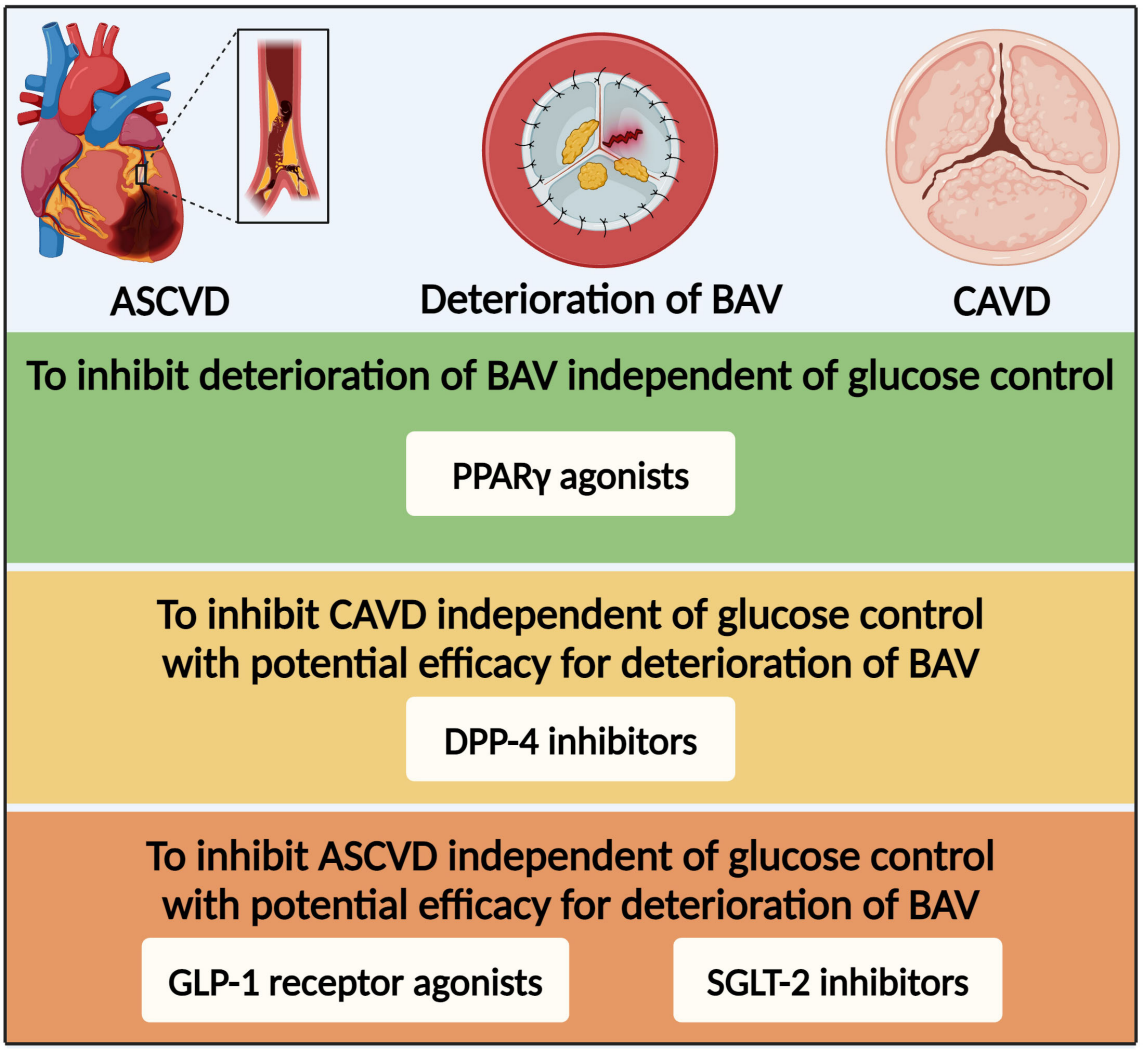
Glucose-lowering medication option for patients with diabetes and deterioration of bioprosthetic aortic valve based on the efficacy for CAVD and ASCVD. ASCVD, atherosclerotic cardiovascular disease; BAV, bioprosthetic aortic valve; PPARγ, peroxisome proliferator-activated receptor γ; DPP-4, dipeptidyl peptidase-4; GLP-1, glucagon-like peptide-1; SGLT-2, sodium–glucose co-transporter-2.

Once SVD developed into bioprosthetic valve failure, the death rate increased rapidly except in cases of transcatheter valve-in-valve implantation or transthoracic reoperation (203). However, there are currently no medications to prevent or reverse the progression of BAV deterioration. The peroxisome proliferator-activated receptor γ (PPARγ) was a nuclear receptor that participated in various physiological processes as a transcriptional regulator (212). Activation of PPARγ by specific agonists, thiazolidinediones such as pioglitazone, has been widely used for lowering glucose via insulin-sensitizing and pancreatic β-cell preserving effects (213). Apart from glucose-lowering effect, PPARγ agonists have also been found to have anti-atherogenic and anti-inflammatory effects via regulating the expression of related genes (214, 215). Single-cell RNA sequencing analysis found conservation of PPARγ in non-calcified human aortic valve leaflets and activated PPARγ pathway in CD36-positive VECs in hyperlipidemic mice (216). Many vitro and vivo studies have revealed the anti-calcification effect of PPARγ agonists in the degeneration of native aortic valves (30, 80). Moreover, its effect on the deterioration of BAV was assessed in various rat models. In the streptozotocin-induced diabetic rats, pioglitazone led to an inhibition of BAV deterioration, manifested with a lower expression of chondro-osteogenic genes and calcium deposits (217). In the guide wire injury-induced AVS rats as well as hypercholesterolemic and obese rats, systemic PPARγ activation inhibited inflammation and calcification in heterologous aortic valve conduits and seemed to inhibit functional impairment of the implanted aortic valve (218, 219). Overall, based on the protective effects for CAVD and BAV deterioration *in vitro* and vivo, PPARγ agonists are currently one of the promising glucose-lowering medications to inhibit deterioration of BAV probably independent of glucose control (**Figure 5**).

Dipeptidyl peptidase-4 (DPP-4) inhibitors have been widely applied to treat diabetes via inhibiting degradation of GLP-1 to regulate insulin secretion (220). DPP-4 was widely expressed in the cardiovascular tissues and participated in the physiopathologic process of various cardiovascular diseases (221). Although multiple large-scale clinical studies have demonstrated statistical non-inferiority but not superiority for the DPP-4 inhibitors in the primary MACE endpoint (222, 223), recent studies have found that DPP-4 inhibitors showed benefits on various cardiovascular diseases, such as hypertension, CAD, and CAVD (224). *In vitro*, DPP-4 upregulation by nitric oxide deprivation-dependent NF-κB activation resulted in osteogenic differentiation of VICs (225). *In vivo*, DPP-4 inhibitors markedly reduced calcification of aortic valve in eNOS-deficient mice and rabbit fed with cholesterol-enriched diet and vitamin D (225, 226). DPP-4 inhibitors suppressed CAVD by alleviating inflammation, fibrosis, and calcification (225–227). Overall, DPP-4 inhibitors might be able to inhibit CAVD independent of glucose control with potential efficacy for deterioration of BAV.

Over the last decade, the results of numerous large cardiovascular outcome trials in patients with diabetes at high cardiovascular risk with novel glucose-lowering medications, such as sodium-glucose co-transporter-2 (SGLT-2) inhibitors and GLP-1 receptor agonists, have substantially offered more available medications, resulting in brand new evidence-based medical therapy for the management of this population (10). Based on the cardiovascular benefits independent of glucose control, SGLT-2 and GLP-1 receptor agonist inhibitors might be the promising medications with potential efficacy for CAVD or even deterioration of BAV. Thus, further experiments and large-scale clinical studies are essential for verification of the effects of glucose-lowering medications on deterioration of BAV.

## 7 Conclusions

In recent years, the prevalence of diabetes has increased rapidly in patients with CAVD. Except for individual effects of diabetes (e.g., hyperglycemia, AGEs/RAGE pathway, oxidative stress, EndMT, and inflammation), there were multifactorial interactions between those diabetic complications (e.g., hypertension, hyperlipidemia, CKD, ageing), which mutually took part in the initiation and development of CAVD. The prevalence of diabetes in patients undergoing TAVR or SAVR has also increased along with the progression of CAVD. However, clinical outcomes and postoperative complications in diabetes after TAVR or SAVR remained controversial. Compared with non-diabetes, the short-term mortality after TAVR was not significant in diabetes, while the mid-term mortality remained disputable. The mid-term and long-term mortality rates after SAVR were higher in patients with diabetes than non-diabetes, while the short-term mortality remained disputable. There were common worse manifestations with CAD, AKI, heart failure, and SIRS in diabetes undergoing TAVR or SAVR, compared with non-diabetes. The role of diabetes-related obesity paradox in TAVR or SAVR remains disputable. There is a need for RCT and large cohorts with long-term follow-up of diabetes vs. non-diabetes in patients undergoing TAVR or SAVR. Moreover, diabetes and its complications also jointly participated in the deterioration of BAV, leading to increased long-term mortality and various postoperative complications after TAVR or SAVR. Based on the efficacy for CAVD and BAV deterioration *in vitro* and vivo, PPARγ agonists might be the promising glucose-lowering medication to inhibit BAV deterioration independent of glucose control.
